# Supplementary material for: Ionic thermoelectric gating organic transistors
Source: Nat Commun. 2017 Jan 31;8:14214. doi: 10.1038/ncomms14214 (PMC5290323; doi:10.1038/ncomms14214)
Supplement: Supplementary Information — Supplementary Figures 1-7 [file ncomms14214-s1.pdf]

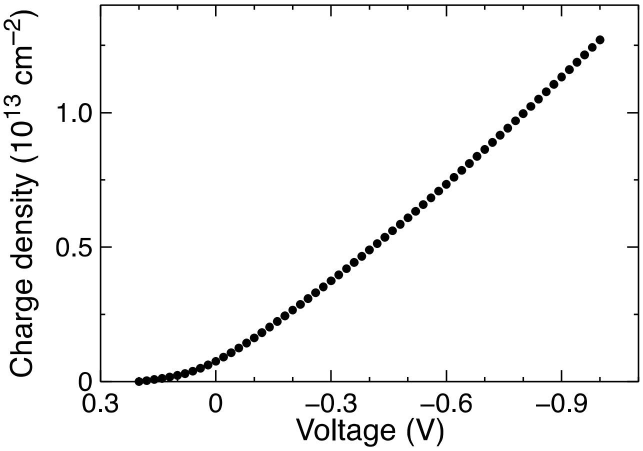

Supplementary Figure 1. Charge carrier density as a function of the DC voltage bias for a P3HT-P(VPA-AA) MIS device with Ti gate metal electrode.

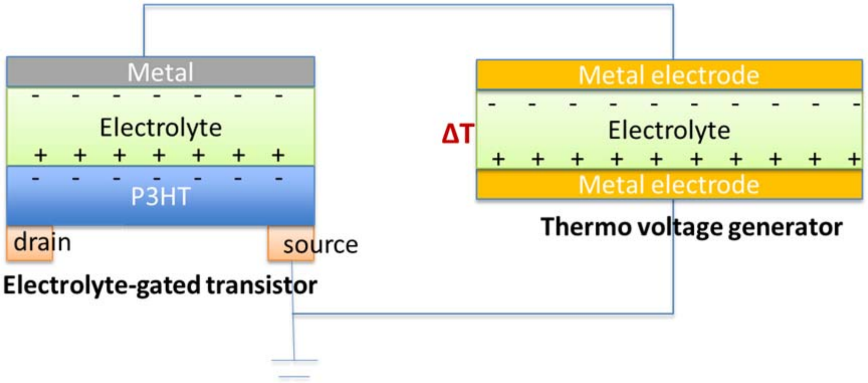

Supplementary Figure 2. The structure of the ionic thermoelectric gated transistor. One electrode (working electrode) of the thermoelectric device is connected to the gate of the transistor, while the other electrode (grounded electrode) is connected to the source and grounded together. When temperature difference applied, the voltage of the gate is the same as the working electrode of the thermoelectric device.

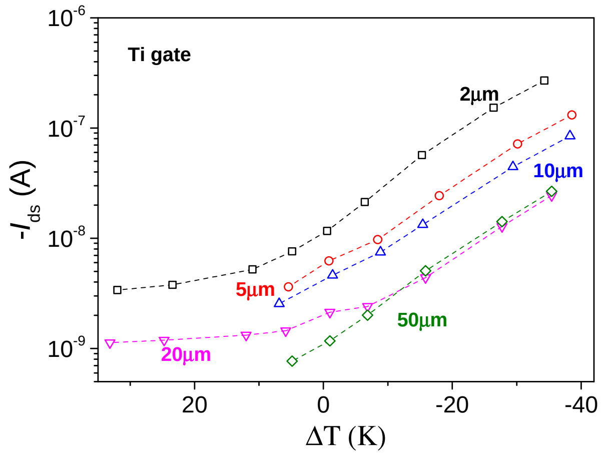

Supplementary Figure 3. Transfer characteristics with different channel length of Ti gate.

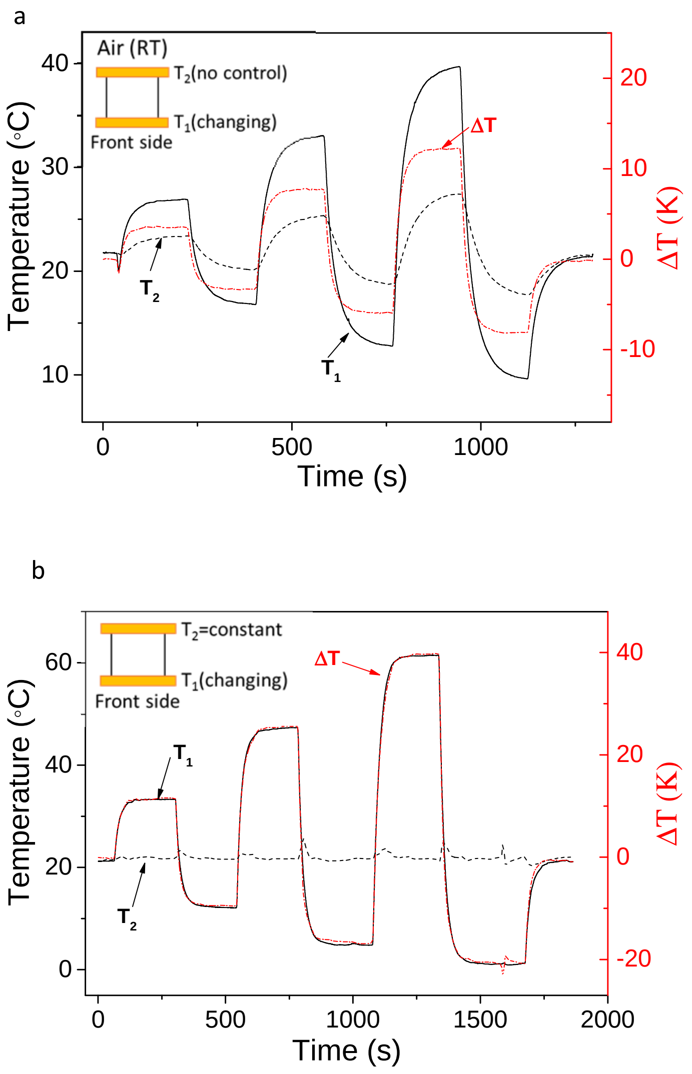

Supplementary Figure 4. The correlation between measured ΔT changing with real temperature. a) ΔT changing with the real temperature of the front side of the device, while the temperature of the other electrode is kept constant. The black is the temperature of the front (T_1) side, and black dashed line is the temperature of the other side (T_2), and the red dashed line is ΔT. b) ΔT changing with the real temperature of one electrode, while the other electrode is kept constant temperature. During this measurement, the temperature of the front side is heated or cooled, while the other side is leaving open without temperature control, ΔT is measured at the same time.

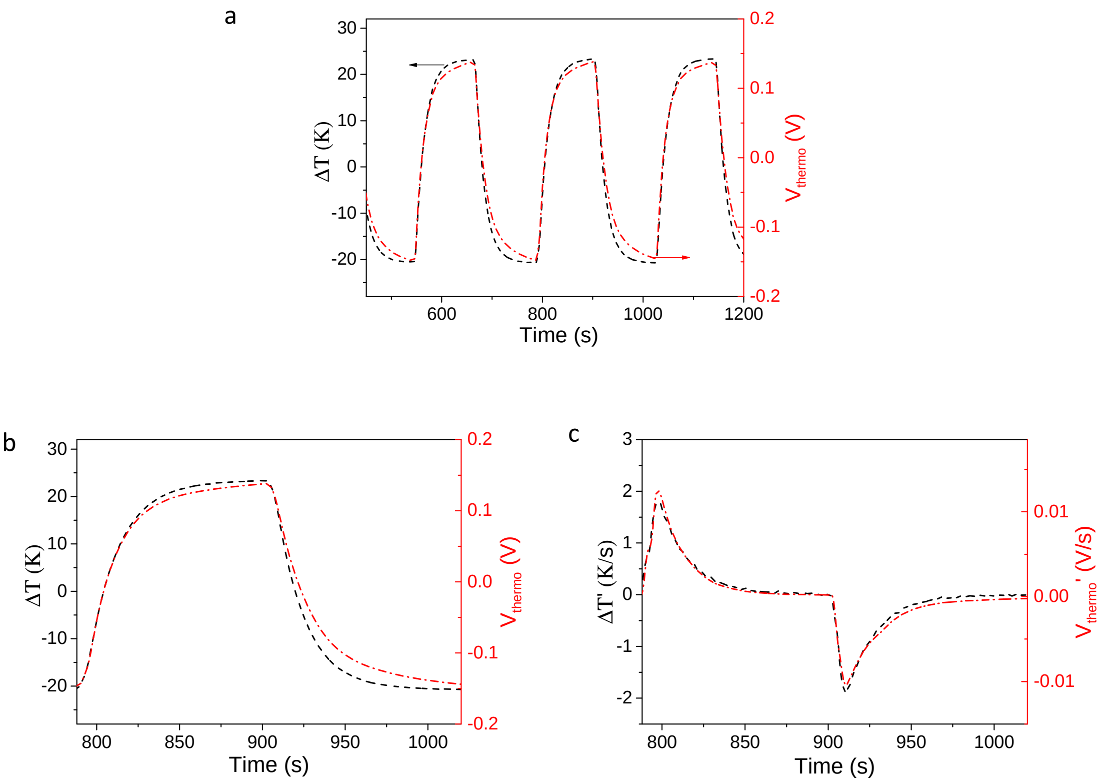

Supplementary Figure 5. a) Measured ΔT across the thermoelectric leg versus time (black dot-dashed line) and the time evolution of V_{thermo} (ref dashed line) b) Detailed zoom in of a). c) the derivation of b), shows the changing rate of ΔT and $V_{thermo.}$

The temperature of the device is controlled by two paltier element same as in the main text. Temperature of both electrodes is measured with thermometer composed of thin gold line.

a

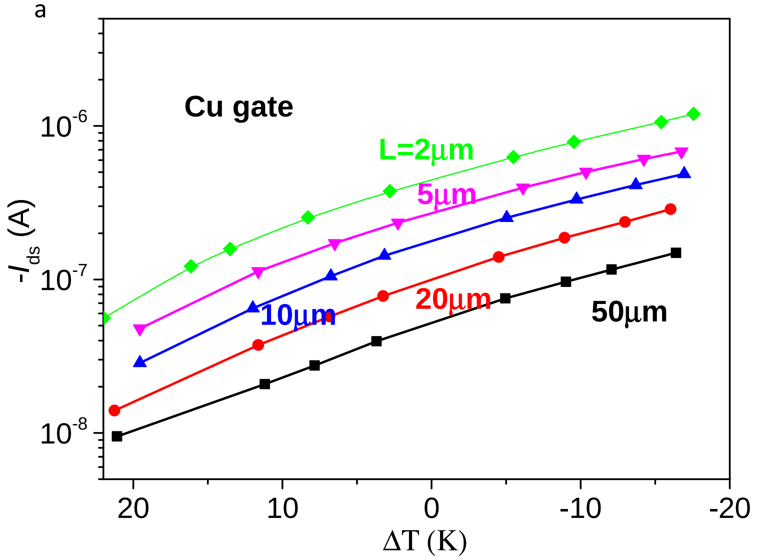

b

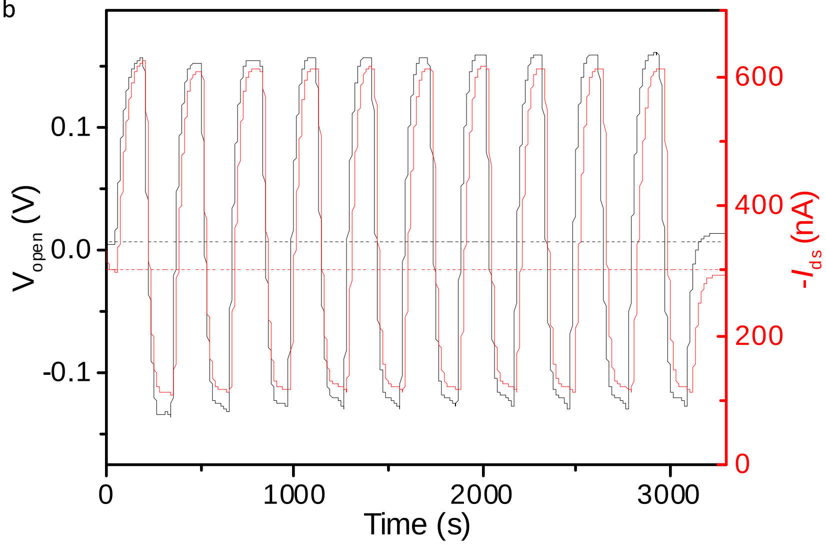

Supplementary Figure 6. (a) Transfer characteristics with different channel length of Cu gate. (b) Multi circles operation of the heat-gated transistor with Cu gate.

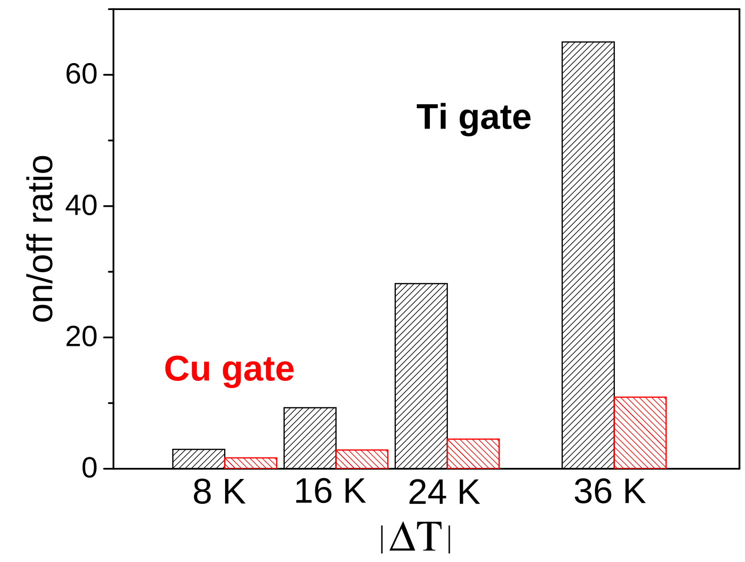

Supplementary Figure 7. The on/off ratio of the thermoelectric gated transistor with Cu and Ti gate metal when the temperature exchanged between the two electrodes. The x-axis represents the absolute temperature difference between the two electrodes.
